# Interactive effects between diet and genotypes of host and pathogen define the severity of infection

**DOI:** 10.1002/ece3.356

**Published:** 2012-08-14

**Authors:** Ji Zhang, Ville-Petri Friman, Jouni Laakso, Johanna Mappes

**Affiliations:** 1 Department of Biological and Environmental Science, Centre of Excellence in Biological Interactions, University of JyväskyläP.O. Box 35, 40014, Jyväskylä, Finland; 2 Department of Biological and Environmental Science, Centre of Excellence in Biological Interactions, University of HelsinkiP.O. Box 65, 00014, Helsinki, Finland

**Keywords:** Genotype-environment interaction, immunocompetence, *Parasemia plantaginis*, *Plantago major*, *Serratia marcescens*, virulence

## Abstract

Host resistance and parasite virulence are influenced by multiple interacting factors in complex natural communities. Yet, these interactive effects are seldom studied concurrently, resulting in poor understanding of host-pathogen-environment dynamics. Here, we investigated how the level of opportunist pathogen virulence, strength of host immunity and the host condition manipulated via diet affect the survival of wood tiger moth *Parasemia plantaginis* (Arctidae). Larvae from “low cuticular melanin” and “high cuticular melanin” (considered as low and high pathogen resistance, respectively) selection lines were infected with moderately and highly virulent bacteria strains of *Serratia marcescens*, while simultaneously manipulating host diet (with or without antibacterial compounds). We measured host survival and food preference before and after infection to test whether the larvae “self-medicate” by choosing an anti-infection diet (*Plantago major*, i.e., plantain leaf) over lettuce (*Lactuca sativa*). “High melanin” larvae were more resistant than “low melanin” larvae to the less virulent strain that had slower growth and colonization rate compared with the more virulent strain. Cuticular melanin did not enhance survival when the larvae were infected with the highly virulent strain. Anti-infection diet enhanced survival of the “high melanin” but not the “low melanin” hosts. Survival was dependent on family origin even within the melanin selection lines. Despite the intrinsic preference for lettuce, no evidence of self-medication was found. These results demonstrate that the relative benefit of host cuticular melanin depends on both diet and pathogen virulence: plantain diet only boosted the immunity of already resistant “high melanin” hosts, and cuticular melanin increased host survival only when infected with moderately virulent pathogen. Moreover, there was considerable variation in host survival between families within both melanin lines suggesting genetic basis for resistance. These results indicate that although melanin is an important predictor of insect immunity, its effect on disease outcomes greatly depends on other interacting factors.

## Introduction

Both the resistance of hosts and the infectivity of pathogens vary considerably in nature (Beldomenico and Begon 2010). Therefore, the severity of infection will crucially depend on the interaction between host and pathogen genotypes (Scholthof 2007; Beldomenico and Begon 2010). In addition to genetic factors (Cotter and Wilson 2002), resistance of the victim depends on its condition, which in turn can depend on the environment. For example, high-quality food can improve the condition and immune defense of individuals (Ojala et al. 2005; Lee et al. 2008; Alaux et al. 2010; Ponton et al. 2011). Moreover, some insects can sequester plant chemicals and directly use them as defensive substances against invading pathogens (Nieminen et al. 2003; Hartmann et al. 2005; Harvey et al. 2005; Baden and Dobler 2009; Singer et al. 2009). On the other hand, environmental factors can also affect pathogen virulence (Johnson 1991; Mekalanos 1992; Friman et al. 2011). Elevated temperature and environmental productivity, for example, can affect the expression of many important bacterial virulence factors (Meyer et al. 2012; Smirnova et al. 2001; Friman et al. 2011).

While some environmental factors are likely to affect only the host or the pathogen, others might have an additive or interactive effect on both sides. For example, diet can influence both host immunity and bacterial infectivity (Ojala et al. 2005; Frost et al. 2008; Lee et al. 2008; Zaborin et al. 2009): simply changing the phosphate concentration of the medium, the death rate of the host can range from 0% to 60% in the nematode-bacteria infection model (Zaborin et al. 2009). Given the complexity of natural communities, it is likely that a range of interactions is important to epidemiological outcomes (Mitchell et al. 2005; Allen and Little 2011; Vale et al. 2011). Most studies to date, however, have manipulated only one variable (host or pathogen genotype or environmental conditions) at a time; as a result, we understand poorly how environmental conditions interact with different host and pathogen genotypes during the infection.

Here, we studied these interactions experimentally with the herbivorous *Parasemia plantaginis* (Arctidae) tiger moth larvae and *Serratia marcescens* bacteria (Friman et al. 2009). The larvae have an orange patch on the dorsal side of an otherwise black body. The larvae are aposematic: the patch is used as a warning signal, and the size of the patch is heritable (Lindstedt et al. 2009). Bigger patches are more effective warning signal for avian predators (Lindstedt et al. 2008). On the other hand, investing in a large orange warning signal decreases the amount of cuticular melanin: the amount of melanin correlates positively with immune responses in many taxa including *P. plantaginis* (Armitage and Siva-Jothy 2005; Siva-Jothy et al. 2005; Friman et al. 2009; Laurentz et al. 2012). In this study, we used *P. plantaginis* larvae from two selection lines for low or high amount of cuticular melanin. Although detailed immunological mechanisms are not understood, the previous experiments have shown that “Low melanin” individuals (with larger orange patches) have weaker pathogen resistance than “high melanin” individuals (with small patches) (Friman et al. 2009).

Larvae from both selection lines were infected with one of two *S. marcescens* strains, either DB11 or ATCC 13880. *S. marcescens*is a natural pathogen of many insect species including larval Lepidoptera (Grimont and Grimont 1978; Sikorowski and Lawrence 1998; Inglis and Lawrence 2001). To simulate natural infection more realistically, all larvae were infected with the bacteria orally instead of injection (Vodovar et al. 2004, 2005). The strain DB11 is highly virulent to nematodes (Pujol et al. 2001) and *Drosophila* (Nehme et al. 2007) and was originally isolated from a dead fruit fly (Flyg et al. 1980). The strain ATCC 13880 was originally isolated from pond water. Thus, we expected it to be less virulent in *P. plantaginis* host compared with strain DB11 because it has no known close evolutionary history with insect pathogens. We also measured the growth and motility of these two bacterial strains to study potential mechanisms of virulence.

To potentially manipulate host condition via diet, the larvae were fed either plantain (*Plantago major*) or lettuce (*Lactuca sativa*) ad libitum. Plantain leaves is used to heal wounds and infections in traditional medicine in many countries (Hetland et al. 2000; Samuelsen 2000). In addition, it has antiinflammatory, antimicrobial, and antitumor effects (Gomez-Flores et al. 2000). Lettuce, on the other hand, has been shown to decrease larval immune response (Ojala et al. 2005).Thus, it is possible that these plant diets have opposing effects on larval immunity, which could result in difference in survival during infection. Alternatively, diets could have different effects on larval growth, development, and other life-history traits (Ojala et al. 2005; Laurentz et al. 2012), which could also affect larval survival during infection.

Parasites or pathogens can induce infected individuals to adopt a diet that helps them to fight the infection (Clark and Russell Mason 1988; Huffman et al. 1996; Christe et al. 2003; Milan et al. 2012). Self-medication has been recently demonstrated also among caterpillars (Krischik et al. 1988; Lee et al. 2006; Povey et al. 2009; Singer et al. 2009). Therefore, we also tested whether *P. plantaginis* larvae changed their feeding preference after the bacterial infection.

We hypothesized that “high melanin” individuals feeding on plantain would have the lowest infection mortality, if diet and host resistance additively boost host immunity. However, this effect could depend on the level of pathogen virulence, and similarly, diet could have different effects on larval survival depending on the level of host resistance (amount of melanin) and host genetic background. As a result, the relative importance of each experimental manipulation could depend on how it interacts with other factors.

## Material and Methods

### Host rearing, bacterial infection, and food preference tests

Selection lines for “high melanin” (small orange patch) and “low melanin” (large orange patch) in the *P. plantaginis* larvae were established in 2004; 51 families were used to set up the selection lines by applying a truncated family selection protocol (Lindstedt et al. 2009). In this experiment, we used a total of 335 individuals originating from 22 different families (Table 1). We used 10 families from high melanin selection line (6–27 individuals per family, 147 larvae in total), and 12 families from low melanin selection line (13–19 individuals per families, 188 larvae in total). Larvae were isolated from a laboratory stock after they were reared on dandelion (*Taraxacum* sp.) in constant laboratory conditions (Friman et al. 2009).

**Table 1:** Summary of the wood tiger moth larvae used in the experiment

Selection line	Diet	Bacterial treatment	*n*
High melanin (*n* = 147)	Plantain (*n* = 71)	NC	21
		ATCC 13880	24
		DB11	26
	Lettuce (*n* = 76)	NC	24
		ATCC 13880	25
		DB11	27
Low melanin (*n* = 188)	Plantain (*n* = 88)	NC	26
		ATCC 13880	32
		DB11	30
	Lettuce (*n* = 100)	NC	31
		ATCC 13880	35
		DB11	34

NC, negative control.

The *S. marcescens* strain ATCC 13880 was obtained from American Type Culture Collection, while the *S. marcescens* strain DB11 was kindly provided by Prof. Hinrich Schulenburg. We prepared the bacterial inoculums by first growing both strains overnight on LB agar plates. After 24-h growth at 25°C, sterile loops (VWR) were used to streak and dilute bacterial cells to sterile water to optical density of 1.0 (Bioscreen C spectrophotometer (Oy Growth Curves Ab Ltd, Helsinki, Finland), OD 420–580 nm, wide band option) equalling approximately 5.4 × 10^8^ (ATCC 13880) and 5.8 × 10^8^ (DB11) bacterial cells/mL. Sterile water was used as a negative control.

When the larvae were 3 weeks old, we separated them individually to 9 cm Petri dishes and permanently changed their diet to either lettuce or plantain for the rest of the experiment. It is possible that mere switch from one food plant to another could be stressful, making it difficult to separate stress effects from effects of food per se. However, in this experiment, we wanted to study the short-term effects of different plant species on larval survival and thus wanted to exclude the long-term developmental effects of different plant diets. As a result, we reared larvae before infection on dandelion (to ensure similar handling for all treatments). To avoid a confounding effect of body mass on survival, we distributed the “high melanin” and “low melanin” larvae to the treatments evenly according to the body mass (Two-way ANOVA [analysis of variance], *P* > 0.353 for all pair-wise comparisons between all treatments). After 2 days on the new diet, we performed a pre-infection diet preference test. All the larvae were food-deprived for 24 h before offering fresh leaves (cut to 2 × 2cm squares) of both lettuce and plantain. The leaves were put on a moisturized filter paper to prevent drying. We measured the proportion of both leaves consumed after 24 h. The post-infection food preference test was performed twice, 24 and 72 h after the infection as described above (excluding food-deprivation).

In most bacterial infection studies, bacteria are injected with a needle into the body cavity of the host (septic injury model; Vodovar et al. 2004). In order to simulate natural infection more realistically, all larvae in the present experiment were infected with the bacteria orally (Vodovar et al. 2004, 2005). After the pre-infection food preference test, the larvae were presented with 20 *μ*L of either bacterial inoculum (DB11 or ATCC 13880) or sterile water. The larvae were monitored until they had drunk the whole inoculum. After the infection and the post-infection food preference test, fresh lettuce or plantain leaves were added on to the Petri dishes ad libitum, according to the diet treatment. We recorded larval survival twice a day for the following 4 weeks. The larvae were reared at 25°C during the infection experiment.

### Bacterial growth ability and motility measurements

Bacterial growth indicates how efficiently bacteria can turn resources into biomass, which probably correlates with the ability to reproduce within the hosts (host exploitation rate; Harrison et al. 2006). We assessed resource use ability of the *S. marcescens* strains as short-term (24 h) maximum growth rate and maximum density in vitro as follows: small bacterial inoculums (<0.0002% of the maximum population size) were added to fresh bacterial culture medium (hay extract) at a low initial density (Friman et al. 2008). Maximum growth rates and population sizes were determined from biomass growth data recorded for 96 h at 10 min intervals (Bioscreen C spectrophotometer, 420–580 nm optical density). Three different resource concentrations were used to estimate the growth parameters: low-, intermediate- and high-resource concentrations (containing 0.53, 1.07- and 2.15 mg L^−1^ final concentration of plant detritus, respectively). Ten replicate measurements were used for both strains in all resource concentrations.

High motility can increase bacterial virulence through enhanced host colonization ability (Johnson 1991; Josenhans and Suerbaum 2002; Lane et al. 2007). Bacterial motility assays were performed in vitro by stabbing trace amount (2 *μ*L) of each bacterial strain with sterile loops (VWR) on the center of semi-fluid NB agar plates containing 0.7% of agar (Friman et al. 2009). The motility of strains was determined as the area (mm^2^) bacteria were able to colonize within 24 h (*N* = 10 for both bacterial strains). All bacterial trait measurements were conducted at 25°C.

### Statistical analyses

We analyzed larval survival with the Cox regression survival analysis and Log-rank statistics. Initially, we used a full Cox regression model where larval survival was explained with following variables: diet, bacterial treatment (bacterial strain type and water), initial body mass, and melanin selection line of the larvae. This model showed a significant difference in survival between control and both bacterial strains (see Results). In the next analysis, we excluded the larvae from water control treatment to directly compare the effect of bacterial strains on larval survival. By adding and subtracting all possible interactions one by one, we found that only the initial body mass × melanin selection line, and initial body mass × diet interactions improved the model significantly (*χ*^2^ = 4.577, *P* = 0.032, Table S1). As a result, the final model included the main effects of diet, bacterial strains, initial body mass, melanin selection line of the larvae, and initial body mass × melanin selection line and initial body mass × diet interactions as explaining factors. To separate the effect of family from the effect of melanin selection line, we also analyzed the effect of above-mentioned factors separately within both selection lines.

We compared the relative consumption of lettuce versus plantain in two ways. First, we analyzed the consumption of different diets before and after the infection separately in each time point (0, 24, and 72 h from infection). In these models, larval food consumption was explained by consumption of the given diet (lettuce or plantain), melanin selection line, diet type, infection treatment, and their interactions. Larval weight was used as covariate and family was nested under the selection line and included in the model as a random factor. Second, we analyzed if the food preference changed through time (proportion of lettuce consumption – proportion of plantain consumption) between pre- and post-infection food preference tests using repeated-measures ANOVA. Two-way ANOVA was used to compare the growth and the motility of *S. marcescens* bacterial strains. All analyses were performed with SPSS v. 20 (IBM, International Business Machines Corp., Armonk, New York).

## Results

**Figure 1:**
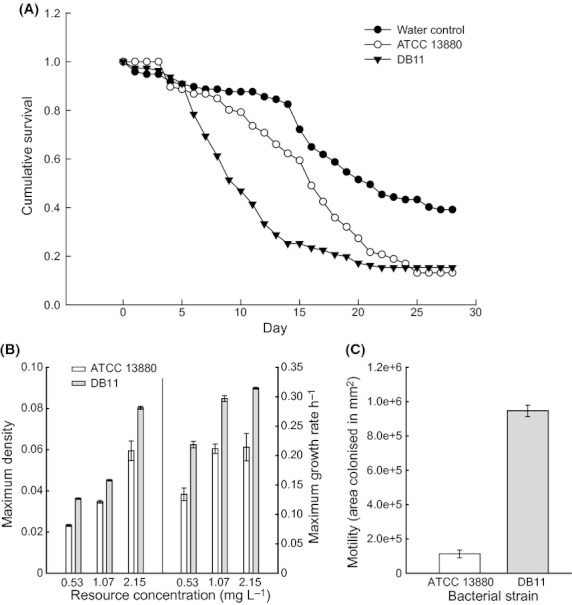
(A) Survival curve for larvae infected with water (filled circles), strain ATCC 13880 (open circles) and strain DB11 (filled triangles). (B) maximum population sizes and growth rates of strain ATCC 13880 (white bars) and DB11 (gray bars) measured in low-, intermediate- and high-resource concentrations. (C) The motility of ATCC 13880 (white bars) and DB11 (gray bars). Error bars in (B) and (C) denote ±1 SEM.

We found that both *S. marcescens* strains decreased larval survival compared with water control group (DB11: *β* = 2.138, *P* < 0.001; ATCC 13880: *β* = 1.668, *P* = 0.015, Fig. 1A), Moreover, strain DB11 killed the larvae faster compared with strain ATCC 13880 (*β* = 0.663, *P* = 0.006). At the bacterial trait level, the higher virulence of strain DB11 was connected to more efficient growth (maximum population size: *F*_1, 53_ = 564.3, *P* < 0.001, maximum growth rate: *F*_1, 53_ = 199.8, *P* < 0.001; differences significant in all tested resource concentrations, *P* < 0.001 in all pair-wise comparisons, Fig. 1B) and higher motility (*F*_1, 9_ = 451.8, *P* < 0.001, Fig. 1C).

**Figure 2:**
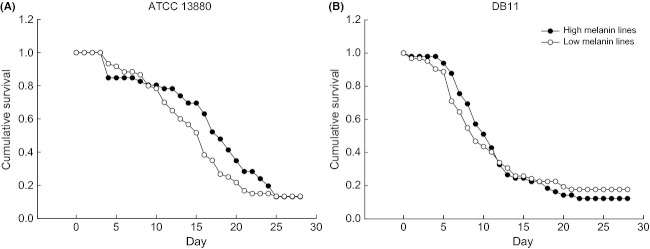
Survival curve of “high melanin” larvae (filled circles) and “low melanin” larvae (open circles) when infected with *Serratia marcescens* strain ATCC 13880 (A) or DB11 (B).

Larvae from the “high melanin” selection line survived better than larvae from the “low melanin” selection line (*β* = −1.654, *P* = 0.010), but this was only true when they were infected with the low virulence strain ATCC 13880 (*β* = −0.634, *P* = 0.007, Fig. 2A).The difference between the melanin selection lines (host genotypes) vanished when the more virulent strain DB11 was used for infection (*β* = −0.200, *P* = 0.363, Fig. 2B). Larger larvae had better survival in general (*β* = −0.045, *P* < 0.0001). The effect of larval weight was the same in all treatments.

**Figure 3:**
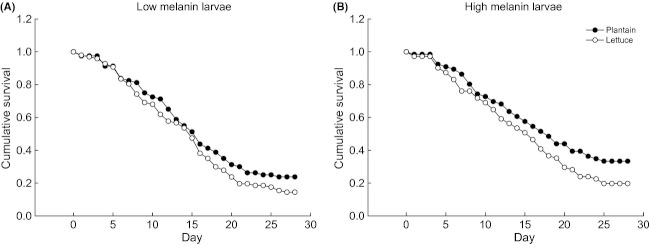
Survival curve of larvae from low melanin (A) and high melanin (B) selection lines when fed with plantain (filled circles) or lettuce (open circles).

The main effect of diet in the water control group was close to significant (*β* = 0.103, *P* = 0.067), suggesting that plantain diet could increase larval survival in general. However, feeding on common plantain enhanced larval survival only within the “high melanin” selection line (*β* = −0.513, *P* = 0.013, Fig. 3B); diet had no effect on larval survival within the “low melanin” selection line (*β* = −0.126, *P* = 0.467, Fig. 3A).

Interestingly, the family origin strongly affected larval survival (Wald = 48.633, *P* < 0.0001). However, no interaction between family and bacterial strain was found (Wald = 16.738, *P* = 0.541). This indicates that the effect of family on host survival was similar within both selection lines, and was not affected by the type of bacterial strain.

**Figure 4:**
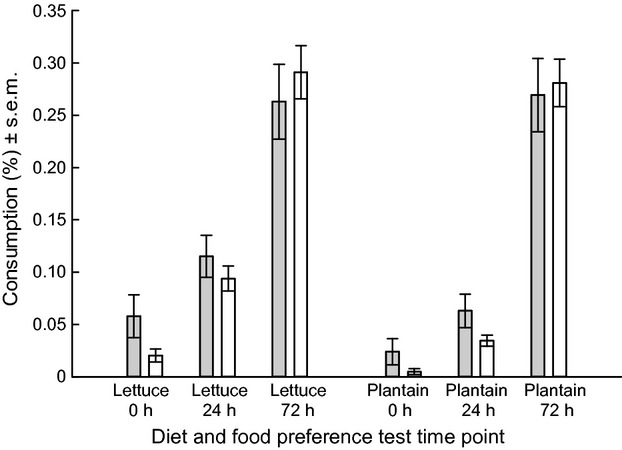
Food preferences of the larvae before and after bacterial infection. The plantain consumption (gray bars) and lettuce consumption (white bars) are shown separately for each bacterial strain. Error bars denote ±1 SEM.

We did not find evidence of self-medication. Larvae consumed more lettuce compared with plantain before (*F*_1, 640_ = 5.7, *P* = 0.017) and after 24 h (*F*_1, 635_ = 19.1, *P* < 0.001) of bacterial infection, while no difference was observed after 72 h of infection (*F*_1, 633.8_ = 0, *P* = 0.9). However, food preference was similar regardless of bacterial infection treatment (no other significant main effects or interactions were found; all *P* > 0.05). Moreover, the larval food preference did not change (consumption of lettuce relative to plantain) before or after infection (RANOVA: the effect of time and all its interactions were non-significant, *P* > 0.2 in all cases; the effects of diet, bacterial strain, melanin signal line, and all their interactions non-significant, *P* > 0.15 in all cases, Fig. 4).

## Discussion

Our goal was to study comprehensively how the level of host resistance, pathogen virulence, and diet interact to determine the outcome of a bacterial infection. As expected, larvae with high level of cuticular melanin level survived best from infection. Even though larvae preferred lettuce to plantain for a short period after diet switch (∼24 h), no evidence for self-medication was found. However, plantain diet boosted the survival of larvae from the high, but not from the low, melanin selection lines. Interestingly, the host genotype (selection line) and diet only had an effect on larval survival when the less virulent strain ATCC 13880 was used for the infection. Together, these results suggest that both the quality of the host's environment and host genotype can decrease the success of a moderately virulent, but not a highly virulent, bacterial pathogen. As a result, self-medication behavior and genetically mediated host resistance are more likely to evolve in the presence of less virulent pathogens.

*Serratia marcescens* strain DB11 caused higher host mortality compared with strain ATCC 13880 (Fig. 1A). This result is in accordance with our expectations due to the strains’ different origin; bacteria isolated from aquatic environment are likely to be less virulent because they share no close evolutionary history with insect hosts. At the mechanistic level, higher DB11 virulence was connected to more efficient growth and motility measured in vitro (Figs. 1B, C). Bacterial growth rate is an indicator of efficient host exploitation rate (Johnson 1991; Meyer et al. 2012; Harrison et al. 2006; Friman et al. 2009). DB11 had both higher maximum growth rate and maximum density in all resource concentrations we used in the in vitro measurements. Together, these results suggest that high bacterial competitive ability could be important for bacterial fitness in both external natural reservoirs and within the host (Walther and Ewald 2004). Growth ability also correlated positively with motility, which can increase virulence by improving host colonization efficiency (Johnson 1991; Pujol et al. 2001; Josenhans and Suerbaum 2002; Lane et al. 2007; Malik-Kale et al. 2007). For example, according to previous studies, motility is needed for successful infection in both *Drosophila* (Nehme et al. 2007)and *Nematode* (Pujol et al. 2001) hosts. Moreover, the non-motile and closely related DB140 mutant strain is non-virulent in nematodes (Pujol et al. 2001), while less motile ATCC 13880 genotypes are less virulent in *P. plantaginis* (Friman et al. 2009, 2011). Unfortunately, our experimental setting is not adequate to estimate the relative importance of these two potential virulence mechanisms. However, in more general perspective, these results suggest that bacterial virulence can correlate positively across different host organisms regardless of its evolutionary origin, i.e., bacterium isolated from Diptera host is also virulent in Lepidoptera host (Jander et al. 2000).

*Parasemia plantaginis* larvae from the “high melanin” selection line were more resistant than the “low melanin” individuals against the less virulent ATCC 13880 bacterial strain (Fig. 2). Melanization and phagocytosis play a major role in eliminating bacterial infection in insects (Hillyer et al. 2003; Nappi and Christensen 2005) by killing most of the invading bacteria (Haine et al. 2008; Schneider and Chambers 2008). High cuticular melanin content often correlates positively with insects’ ability to resist parasites and pathogens (Nappi et al. 1995; Wilson et al. 2001; Cotter et al. 2004) because it is connected to high phenoloxidase (PO) activity (Rowley and Brooy 1986): a major humoral immune defense cascade in insects, which is expressed and regulated in response to the presence of non-self in the haemocoel (Soderhall 1982). Interestingly, there was no benefit of “high melanin” against the more virulent strain suggesting that PO activity-based defense has its limits. For example, it is possible that when the pathogens reproduce fast (which is the case with strain DB11), hosts might not be fast enough in mounting immune responses that require an activation period. Diet quality can also greatly affect the physiological condition and immunocompetence of hosts (Fellous and Lazzaro 2010; Laurentz et al. 2012). We found that plantain diet increased larval survival only within more resistant, “high melanin” selection line (Fig. 3). This is surprising because plantain extracts have antibacterial effects against both Gram-positive and Gram-negative bacteria (Gomez-Flores et al. 2000). Moreover, Plantain diet helps mice to fight systemic infection of *Streptococcus pneumoniae* by stimulating the innate immune system (Hetland et al. 2000), while Plantain extracts can also activate macrophages and affect the lymphocyte proliferation (Gomez-Flores et al. 2000). In addition to direct immunological effects, Plantain leaves can contain as much as 15% of protein (Mohamed et al. #b^400^), which is approximately 10 times the concentration in *Lactuca sativa* leaves (USDA Nutrient Database; http://www.nal.usda.gov/fnic/foodcomp/search/). High protein diet can induce cuticular melanin production, which is correlated with anti-infection activity (Lee et al. 2008; Cotter and Kilner 2010). As only “high melanin” larvae benefited from plantain diet, it seems that instead of increasing survival by improving larval condition in general, the plantain diet interacts with the host immune system. If this was due to plantain diet's positive effect on host resistance or tolerance, or direct negative effect on the parasite, remains still unclear. It is notable that larval survival in the water treatment group was quite poor compared with previous studies (Friman et al. 2009, 2011). One explanation could be the abrupt diet switch from dandelion to plantain or lettuce after the first 3 weeks of larval development. As individuals in all treatments switched diet from dandelion to lettuce or plantain, the results were unlikely biased by the diet-switching. Most importantly, larval survival was clearly higher in bacterial treatments compared with the water control, which shows that bacterial infection increased larval mortality.

Recent findings suggest that insect hosts can change their diet toward medicating plants after infection (Singer et al. 2009).We found intrinsic preference for lettuce, which, however, vanished soon after the diet switch (no difference after 72 h of infection; Fig. 4). Furthermore, all larvae favored lettuce over plantain regardless of whether they were infected with bacteria or not. As a result, this short-term diet preference was unlikely to be connected to self-medication. One potential explanation could be starvation-induced dehydration, which might lead preference for lettuce that has relatively high water concentration. Self-medication can also be trans-generational, and directed toward offspring instead of the infected parent (Lefevre et al. 2010, 2012). Thus, it is possible that the 3-day interval after the infection was too short to observe potential food preference in *P. plantaginis*. Alternatively, complementary diet where larvae consume both medicating and normal growth-enhancing plants could result in highest survival (Ojala et al. 2005). For example, for polyphagous insects, it is often energetically costly to sequester plant chemicals (Berenbaum and Zangerl 1993; Despres et al. 2007; Lindstedt et al. 2010). Thus, while medicating plants could provide direct benefits against pathogens, excessive ingestion of defensive chemicals could reduce larval survival (Singer et al. 2009).

Interestingly, family was one of the most significant determinants for larval survival during the infection, which may be of evolutionary importance. First, it shows that despite artificial selection for more cuticular melanin, within-line genetic variation was still considerably high. Second, it shows that some families resist infection better as a whole, or that some host genotypes cope better with certain bacterial strains. Because we did not find any evidence of family by strain interactions, our results support the first hypothesis of “generally superior genotypes”. This finding, however, leads to a new puzzle: why are weak host-genotypes not wiped out by natural selection? One likely explanation could be a trade-off between host resistance and other fitness traits (Kraaijeveld and Godfray 1997) that favored weakly resistant genotypes in the absence of pathogens.

In conclusion, our study shows that both host resistance and pathogen virulence, and the diet, are all important in determining the outcome of bacterial infection. More specifically, our results demonstrate that the high amount of cuticular melanin increases the survival of *P. plantaginis* moth larvae, and that the medicating plantain diet enhances only the survival of already more resistant, melanic larvae. These results suggest that although melanin is an important predictor of insect immunity, its effect on disease outcome will greatly depend on the three-way interactions between diet and genotypes of both host and pathogen.

## Supporting Information

**Table S1.** Cox regression model fitting.
