# Supplementary material for: Interactive effects between diet and genotypes of host and pathogen define the severity of infection
Source: Ecol Evol. 2012 Aug 14;2(9):2347–56. doi: 10.1002/ece3.356 (PMC3488684; doi:10.1002/ece3.356)
Supplement: Supplementary file 1 [file ece30002-2347-SD1.doc]

Table 1. Cox regression model fitting.

| Model | Full model -2 Log Likelihood | Partial model -2 Log Likelihood | X^2^ | Sig. |
| --- | --- | --- | --- | --- |
| D + B + I + M + D×M | 1735.115 | 1735.395 | 0.280 | 0.597 |
| D + B + I + M + D×B | 1735.006 | 1735.395 | 0.777 | 0.378 |
| D + B + I + M + D×I | 1734.196 | 1735.395 | 2.397 | 0.122 |
| D + B + I + M + B×M | 1734.391 | 1735.395 | 2.007 | 0.157 |
| D + B + I + M + B×I | 1735.273 | 1735.395 | 0.243 | 0.622 |
| D + B + I + M + I×M | 1731.927 | 1735.395 | 6.936 | 0.008* |
| D + B + I + M + I×M + D×M | 1731.624 | 1731.927 | 0.605 | 0.437 |
| D + B + I + M + I×M + D×B | 1731.652 | 1731.927 | 0.550 | 0.458 |
| D + B + I + M + I×M + B×M | 1730.971 | 1731.927 | 1.912 | 0.167 |
| D + B + I + M + I×M + B×I | 1731.824 | 1731.927 | 0.206 | 0.650 |
| D + B + I + M + I×M + D×I | 1729.638 | 1731.927 | 4.577 | 0.032* |
| D + B + I + M + I×M + D×I + D×M | 1728.828 | 1729.638 | 1.621 | 0.203 |
| D + B + I + M + I×M + D×I + D×B | 1729.433 | 1729.638 | 0.410 | 0.522 |
| D + B + I + M + I×M + D×I + B×M | 1728.899 | 1729.638 | 1.478 | 0.224 |
| D + B + I + M + I×M + D×I + B×I | 1729.565 | 1729.638 | 0.147 | 0.701 |

Abbreviations in the model column: D, diet; B, bacterial treatments; I, initial body mass; M, melanin selection line of the larvae. The best fitting model is underlined. * P<0.05.
